# Method comparisons, influence of the number, distribution and range of samples on performance claims

**DOI:** 10.1155/S1463924681000503

**Published:** 1981

**Authors:** D. Burnett, H. M. Barbour, T. F. Woods

**Affiliations:** Departments of Clinical Biochemistry Queen Elizabeth II Hospital Welwyn Garden City and St. Albans City Hospital St. Albans, Herts UK


Because of the difficulties associated with obtaining patient samples and the labile nature of some analytes, manufacturers will always require the assistance of clinical chemistry laboratories in the establishment of performance claims, but our experience suggests that this work should not be undertaken lightly by laboratories and that manufacturers would be advised to assess the resources of any chosen site carefully before proceeding.

ACKNOWLEDGEMENTS
The authors would like to acknowledge the help of Kodak in supplying instruments and materials used in this investigation. We have valued comment from Drs R.B. Coolen and S. Reynolds and extensive secretarial help from Mrs Rosemary Jones during the preparation of this paper.

REFERENCES

[1] PSEP-2, PSEP-3, PSEP-4. Draft documents by the Instrument Evaluation Sub-committee, Evaluation Protocols Area, National Committee for Clinical Laboratory Standards, 771 E.Lancaster Avenue, Villanova, PA 19085, 1978

[2] Curme, H.G., Columbus, R.L., Dappen G.M. *et al* (1978), *Clinical Chemistry*, **24**, 1335

[3] Spayd, R.W., Bruschi, B., Burdick, B.A. *et al* (1978), *Clinical Chemistry*, **24**, 1343

[4] Kodak EKTACHEM GLU/BUN Analyser - Operators Manual (Eastman Kodak Company, Rochester NY)

[5] Wakkers, P.J.M., Hellendoorn, H.B.A., Op De Weegh, G.J. and Heerspink, W. (1975) *Clinica Chimica Acta*, **64**, 173

[6] Westgard, J.D., de Vos, D.J., Hunt, M.R., Quam, E.F., Garber, C.C. and Carey, R.N. (1978) *American Journal of Medical Technology*, **44**, 552 (1978)

[7] Davis, R.B., Thompson, J.E. and Pardue, H.L. (1978) *Clinical Chemistry*, **24**, 611 (1978)

[8] Burnett, D., Barbour, H.M. and Woods, T.F., Method Comparisons, influence of the number, distribution and range of samples on performance claims *Journal of Automatic Chemistry*, this issue, p.178.

[9] Garber, C.C., Westgard, J.O., Milz, L. *et al* (1979), *Clinical Chemistry*, **25**, 1730.

[10] Introduction to Statistical Analysis, Dixon, W.J., Massey, F.J., 3rd edition (McGraw-Hill, New York 1969), Table A-16, p.534-5

### Method comparisons, influence of the number, distribution and range of samples on performance claims

**D. Burnett, H.M. Barbour and T.F. Woods**

*Departments of Clinical Biochemistry, Queen Elizabeth II Hospital, Welwyn Garden City and St Albans City Hospital, St. Albans, Herts, UK.*

### Introduction

The previous paper [2] described two method comparison studies which followed the guidelines of the National Committee for Clinical Laboratory Standards protocol PSEP-4, comparison of methods experiment [1]. The Kodak Ektachem analytical system for urea and glucose was compared with Technicon AutoAnalyzer I methodologies. Two hundred patient samples distributed according to PSEP-4 guidelines were analysed in duplicate by the test and comparative methods. Twice the minimum recommended number of patient samples were used in order to study the effect of sample size above as well as below the recommended minimum number. The data for glucose is presented and the data modified to produce changes in the sample number, distribution and range.

The estimates of slope, intercept and standard error of the estimate of y (Syx) from linear regression analysis are used in the calculation of the tolerance limits and in estimates of total error at medical decision levels, which provide a basis for manufacturers' performance claims. This paper illustrates the way in which sample number, distribution and range could alter the manufacturers' performance claims and gives an indication of the magnitude of these effects. The methods adopted for detection of outliers in the data can also have a marked effect on the claims made.

### Materials and methods

Experimental methods and materials for glucose have been described previously [2]. The distribution of patient samples recommended for glucose analysis was Group A (<2.8 mmol/l) 10%; B (2.9-6.1 mmol/l) 40%; C (6.2-8.3 mmol/l) 30%; D (8.4-13.8 mmol/l) 10%; and Group E (>13.8 mmol/l) 10%. The information in the draft version of the PSEP-4 protocol contained a misprint and groups for glucose were given as A (10%), B (40%), C (20%, D (10%) and E (10%). In our experiment 20% of samples were collected in Group E. However, the recommended distribution and our distribution have been compared with other possible distributions for one hundred samples by data modification described below.

The equations for linear regression analysis were those given in Davies *et al* [3]. Modification of the original data base of two hundred samples analysed in duplicate by test and comparative method is described below.

### Range of samples

The results were divided into their five separate groups (A-E) and modified data sets for linear regression analysis provided by increasing range from low concentrations, A, AB, ABC, ABCD, ABCDE and from high concentrations, E, DE, CDE, BCDE, ABCDE and from mid concentrations, C, BCD, ABCDE.

**Table 1. Distribution of 100 samples prepared from the original data set of 200**

| | Range category (%) | | | | |
|---|---|---|---|---|---|
| | A | B | C | D | E |
| $c_1$ | 10 | 60 | 10 | 10 | 10 |
| $c_2$ | 20 | 40 | 20 | 10 | 10 |
| $c_3$* | 10 | 40 | 30 | 10 | 10 |
| $c_4$** | 10 | 40 | 20 | 10 | 20 |
| $c_5$ | 20 | 20 | 20 | 20 | 20 |
| $c_6$ | 10 | 10 | 20 | 20 | 40 |

* Distribution recommended in PSEP-4
** Distribution used in original method comparison study [2].

### Number of samples

In order to reduce the data from 200 paired duplicate sample analyses to 150, 100 and 50, the samples were first ranked in ascending order based on the mean value of the duplicate analyses by the comparative method. The samples were then numbered in sets of four in ascending order 1, 2, 3, 4; 1, 2, 3, 4 etc. Consequently, if 150 samples were required, the first three samples in each set were extracted, if 100 samples were required the first two samples in each set were extracted, and so on.

### Distribution of samples

From the complete data of 200 paired duplicate sample analyses, six sets of 100 paired duplicate samples could be prepared using the approach described above. The distributions prepared and their designations are given in Table 1. $C_3$ is the distribution recommended by the protocol and $C_4$ the distribution which forms the whole data base in this study.

### Single analyses of samples

Each sample was analysed in duplicate by the test method ($Y_1Y_2$) and the comparative ($X_1X_2$) method and therefore four possible combinations of single rather than duplicate analyses could be prepared:

$X_1\ Y_1; X_2\ Y_1; X_1\ Y_2$ and $X_2\ Y_2$.

### Detection of outliers

Tests for detection of outliers and exclusion results outside the range of each method were used as recommended in PSEP-4 and discussed more fully in a previous publication.

### Results

The whole data base as an X/Y plot with the comparative method as the independent variable X was illustrated in a previous paper [2]. Visual inspection reveals no obvious non-linearity in the data. Figure 1 shows the same data with the comparative method as the independent variable but the vertical axis being the bias of each individual test value from the comparative method (Y-X). Each test and comparative method value is the mean of duplicate determinations and the range of groups A to E is indicated.

Table 2 gives the linear regression estimates and Table 3 calculations of bias, tolerance limits and total error at a medical decision level of 6.6 mmol/l for those data sets which might be realistically encountered in an experimental situation.

Data sets $a_1$ - $a_{11}$ relate to the range of samples, $b_1$ - $b_4$ to number of samples, $c_1$ - $c_6$ to the distribution of samples,

**Table 2. Linear regression data on different data sets**

| Designation of data set | No of samples | Groups represented | Slope | SD | Intercept | SD | Syx | r | $\frac{SDx}{Syx}$ | $\bar{x}$ | Greater than 3.5 times Syx |
|---|---|---|---|---|---|---|---|---|---|---|---|
| a1 | 20 | A | 1.005 | 0.062 | −0.119 | 0.119 | 0.184 | 0.9699 | 3.73 | 1.82 | 0 |
| a2 | 100 | AB | 0.957* | 0.019 | +0.004 | 0.087 | 0.278 | 0.9806 | 5.20 | 4.27 | 1 |
| a3 | 140 | ABC | 0.963* | 0.013 | −0.024 | 0.071 | 0.289 | 0.9878 | 6.57 | 5.15 | 1 |
| a4 | 160 | ABCD | 0.979* | 0.009 | −0.099 | 0.057 | 0.295 | 0.9934 | 8.84 | 5.85 | 0 |
| a5 | 40 | E | 0.975 | 0.019 | +0.322 | 0.406 | 0.657 | 0.9927 | 8.36 | 20.47 | 0 |
| a6 | 60 | DE | 0.993 | 0.012 | −0.108 | 0.212 | 0.575 | 0.9961 | 11.30 | 17.21 | 0 |
| a7 | 100 | CDE | 1.000 | 0.007 | −0.260* | 0.105 | 0.487 | 0.9976 | 14.36 | 13.27 | 0 |
| a8 | 180 | BCDE | 0.997 | 0.005 | −0.200* | 0.054 | 0.415 | 0.9981 | 16.16 | 9.55 | 1 |
| a9 | 40 | C | 1.049 | 0.068 | −0.662 | 0.506 | 0.315 | 0.9278 | 2.34 | 7.36 | 0 |
| a10 | 140 | BCD | 0.082 | 0.012 | −0.124 | 0.079 | 0.309 | 0.9904 | 7.29 | 6.43 | 0 |
| a11 | 200 | ABCDE | 0.996 | 0.004 | −0.178* | 0.046 | 0.397 | 0.9983 | 17.02 | 8.77 | 2 |
| b1 | 50 | ABCDE | 0.983* | 0.007 | −0.077 | 0.082 | 0.354 | 0.9986 | 19.20 | 8.78 | 0 |
| b2 | 100 | ABCDE | 0.995 | 0.006 | −0.185* | 0.064 | 0.392 | 0.9983 | 17.30 | 8.72 | 1 |
| b3 | 150 | ABCDE | 1.000 | 0.005 | −0.211* | 0.055 | 0.409 | 0.9982 | 16.55 | 8.77 | 2 |
| b4 | 200 | ABCDE | 0.996 | 0.004 | −0.178* | 0.046 | 0.397 | 0.9983 | 17.02 | 8.77 | 2 |
| c1 | 100 | ABCDE | 0.984* | 0.006 | −0.120* | 0.050 | 0.304 | 0.9983 | 17.57 | 6.91 | 0 |
| c2 | 100 | ABCDE | 0.983* | 0.006 | −0.130 | 0.048 | 0.299 | 0.9985 | 18.34 | 6.91 | 0 |
| c3 | 100 | ABCDE | 0.984* | 0.006 | −0.140* | 0.056 | 0.320 | 0.9981 | 16.35 | 7.44 | 0 |
| c4 | 100 | ABCDE | 0.995 | 0.006 | −0.185* | 0.064 | 0.392 | 0.9983 | 17.30 | 8.72 | 1 |
| c5 | 100 | ABCDE | 0.994 | 0.006 | −0.163* | 0.065 | 0.394 | 0.9884 | 17.65 | 9.05 | 1 |
| c6 | 100 | ABCDE | 0.997 | 0.006 | −0.190* | 0.091 | 0.480 | 0.9981 | 16.32 | 12.50 | 1 |
| d1 | 200 | ABCDE | 0.996 | 0.004 | −0.162* | 0.046 | 0.397 | 0.9983 | 17.04 | 8.74 | 2 |
| d2 | 200 | ABCDE | 0.994 | 0.004 | −0.216* | 0.046 | 0.398 | 0.9983 | 17.01 | 8.80 | 2 |
| d3 | 200 | ABCDE | 0.996 | 0.004 | −0.138* | 0.049 | 0.419 | 0.9981 | 16.14 | 8.74 | 1 |
| d4 | 200 | ABCDE | 0.995 | 0.004 | −0.192* | 0.049 | 0.422 | 0.9980 | 16.01 | 8.80 | 2 |
| d5 | 200 | ABCDE | 0.996 | 0.004 | −0.178* | 0.046 | 0.397 | 0.9983 | 17.02 | 8.77 | 2 |
| e1 | 200 | ABCDE | 0.996 | 0.004 | −0.178* | 0.046 | 0.397 | 0.9983 | 17.02 | 8.77 | 2 |
| e2 | 178 | ABCDE | 1.011 | 0.006 | −0.276* | 0.053 | 0.349 | 0.9966 | 12.00 | 7.25 | 1 |
| e3 | 177 | ABCDE | 1.005 | 0.006 | −0.238* | 0.050 | 0.330 | 0.9968 | 12.30 | 7.18 | 0 |

* $p < 0.05$

Data sets $d_1$ - $d_4$ are based on single rather than duplicate analyses by both methods

$d_1$ - $d_5$ to single rather than duplicate estimations by each method and $e_1$ - $e_3$ to the exclusion of samples outside the range of each method without dilution and to exclusion of outliers. Data sets $a_{11}$, $b_4$, $d_5$ and $e_1$ are identical and are included for ease of comparison in Table 2.

### Discussion

The NCCLS protocol, PSEP-4, states that "Inaccuracy is quantitated by the estimates of bias at various medical decision concentrations, $X_C$, and by estimation of total error at the medical decision concentration closest to the mean of the comparison of methods data". The bias of a test method at concentration $X_C$ is calculated, bias = $Y_C$ - $X_C$ where $Y_C$ is the predicted value at $X_C$ and is given by a + b x c (the estimate of intercept is given by 'a' and of slope by 'b'). Clearly any factors which influence the estimates of slope and intercept are important in this context and the magnitude of the standard deviations of these estimates will determine the confidence which can be attached to them.

The tolerance limits are calculated and used to estimate the expected total error. The tolerance limits for a desired population proportion (p) and specified confidence ($\gamma$) may be calculated at $X_C$ from the equation given in PSEP-4

$$y_C \pm K\ Syx \sqrt{1 + \frac{1}{N} + \frac{(X_C - \bar{x})^2}{\Sigma (X_i - \bar{x})^2}}$$

where K is the appropriate tolerance factor for a normal distribution (K values for $\gamma = 0.99$ $p = 0.95$ are used in this study) and Syx is the standard error of the estimate of y. Tolerance limits are calculated only for the medical decision concentration closest to the mean of the comparison of methods data. Total error is calculated by taking the differences between the tolerance limits and $X_C$ and the absolute value of the largest difference is taken as the estimate of total error.

It can be seen that estimates of slope and intercept will influence the calculation of the predicted value $Y_C$ and that the magnitude of Syx will affect the tolerance limits and total error. The value of K is influenced by the number of samples used.

Previous authors [3,4,5] have drawn attention to the effects of range and numbers of samples on various linear regression parameters. Slope is used in the calculation of $Y_C$ and different estimates of the slope are obtained with changes in range $a_1$ - $a_{11}$ and distribution of data $c_1$ - $c_6$. The confidence attached to the estimates of slope (which decide whether the slope is significantly different from 1.0) is affected randomly in this comparison of methods by range ($a_1$ - $a_{11}$) and increased by numbers of samples ($b_1$ - $b_4$). The use of single ($d_1$ - $d_4$) instead of duplicate ($d_5$) analyses has a negligible effect in this set of data since only four out of 200 duplicate estimations were greater than the interval of 3.27 times the average absolute difference as recommended in PSEP-4. An additionally important advantage of duplicates is their value in the study of precision profiles (Table 4).

The sign and magnitude of the intercept can also be shown to be influenced by range and distribution of data. No definite trend is apparent when range is extended ($a_1$ - $a_{11}$) but when the distribution is altered ($c_1$ - $c_6$) there was an increasing negative intercept related to the changing slope. The difference found between the intercept obtained for duplicate observations ($d_5$) and various combinations of single observations ($d_1$ - $d_4$) has little effect.

Range has no effect on Syx if the error in the data is constant throughout the range chosen for method comparison. Many clinical chemistry assays exhibit an increase in standard deviation with increasing analyte concentration. Precision profiles for glucose on the AutoAnalyzer and Kodak Ektachem show increasing imprecision (Table 4). Syx increases as more high concentration samples are included in the distribution ($c_1$ - $c_6$) and is also a function of range ($a_1$ - $a_8$) (Table 2) with consequent effects on estimates of tolerance limits and total error. Syx, the error about the regression line, is independent of sample size [3] and this is illustrated in Table 2, $b_2$ - $b_4$.

Sample size has very little effect on linear regression parameters but range and distribution can have effects on slope, intercept and Syx. This is illustrated by the values observed for the calculation of total error in Table 3, which combines slope, intercept and Syx. For example, a change in sample size $b_2$, $b_3$ and $b_4$ has less effect on total error than a change in distribution of samples $c_1$ - $c_6$ and in range of samples $a_4$, $a_8$, $a_{10}$ and $a_{11}$.

The establishment of performance claims by manufacturers as described by the NCCLS includes a comparison of

**Table 3. Bias, tolerance limits and total error at a medical decision level of 6.6 mmol/l**

| Designation of data set | No of samples | Groups represented | $\bar{x}$ | $Y_C$ ($X_C$ = 6.6 Barnett) | Bias $Y_C$ - $X_C$ | Tolerance limits | Total error |
|---|---|---|---|---|---|---|---|
| a4 | 160 | ABCD | 5.85 | 6.36 | −0.24 | 5.69 - 7.03 | 0.91 |
| a8 | 180 | BCDE | 9.55 | 6.38 | −0.22 | 5.45 - 7.31 | 1.15 |
| a10 | 140 | BCD | 6.43 | 6.36 | −0.24 | 5.66 - 7.06 | 0.94 |
| a11 | 200 | ABCDE | 8.77 | 6.39 | −0.21 | 5.51 - 7.27 | 1.09 |
| b2 | 100 | ABCDE | 8.72 | 6.38 | −0.22 | 5.46 - 7.30 | 1.14 |
| b3 | 150 | ABCDE | 8.77 | 6.38 | −0.22 | 5.46 - 7.31 | 1.15 |
| b4 | 200 | ABCDE | 8.77 | 6.39 | −0.21 | 5.51 - 7.27 | 1.09 |
| c1 | 100 | ABCDE | 6.91 | 6.37 | −0.23 | 5.65 - 7.09 | 0.95 |
| c2 | 100 | ABCDE | 6.91 | 6.36 | −0.24 | 5.64 - 7.08 | 0.96 |
| c3 | 100 | ABCDE | 7.44 | 6.36 | −0.24 | 5.60 - 7.12 | 1.00 |
| c4 | 100 | ABCDE | 8.72 | 6.38 | −0.22 | 5.46 - 7.30 | 1.14 |
| c5 | 100 | ABCDE | 9.05 | 6.40 | −0.20 | 5.48 - 7.32 | 1.12 |
| c6 | 100 | ABCDE | 12.50 | 6.39 | −0.21 | 5.26 - 7.52 | 1.34 |
| d1 | 200 | ABCDE | 8.74 | 6.41 | −0.19 | 5.53 - 7.29 | 1.07 |
| d2 | 200 | ABCDE | 8.80 | 6.35 | −0.25 | 5.47 - 7.23 | 1.13 |
| d3 | 200 | ABCDE | 8.74 | 6.44 | −0.16 | 5.51 - 7.37 | 1.09 |
| d4 | 200 | ABCDE | 8.80 | 6.38 | −0.22 | 5.44 - 7.32 | 1.16 |
| d5 | 200 | ABCDE | 8.77 | 6.39 | −0.21 | 5.51 - 7.27 | 1.09 |
| e1 | 200 | ABCDE | 8.77 | 6.39 | −0.21 | 5.51 - 7.27 | 1.09 |
| e2 | 178 | ABCDE | 7.25 | 6.40 | −0.20 | 5.62 - 7.18 | 0.98 |
| e3 | 177 | ABCDE | 7.18 | 6.39 | −0.21 | 5.65 - 7.13 | 0.95 |

**Table 4. Imprecision at different analyte concentrations for the comparative (AA1) and test (Kodak) methods**

**Auto Analyzer 1**

| Group | SD (mmol/l) | mean (mmol/l) |
|---|---|---|
| A | 0.055 | 1.82 |
| B | 0.070 | 4.88 |
| C | 0.098 | 7.36 |
| D | 0.133 | 10.71 |
| E | 0.137 | 20.47 |

**Kodak**

| Group | SD (mmol/l) | mean (mmol/l) |
|---|---|---|
| A | 0.029 | 1.70 |
| B | 0.054 | 4.69 |
| C | 0.083 | 7.06 |
| D | 0.099 | 10.43 |
| E | 0.198 | 20.27 |

methods experiment (PSEP-4) to provide information concerning bias and total error,which are derived from linear regression parameters. In our studies we found that range and distribution had the greatest influence on slope, intercept and Syx, whereas the sample numbers studied had little effect on these parameters. It would therefore seem appropriate to define a minimum range of values and suggested distributions for individual analytes and to provide this information in association with performance claims. Careful inspection of graphical presentation of data is of primary importance. The conventional XY plots of data provide the best approach to the detection of non-linearity whereas the presentation given in Figure 1 where the bias of each individual test result from the comparative method is plotted against the value for the comparative method provides a valuable opportunity to evaluate bias between methods at different analyte concentrations particularly as the scale of the Y axis can be expanded as required. It would also seem appropriate to define the medical decision concentration for calculation of tolerance limits and total error and chose the concentration range and distribution to give a mean value approximating to this concentration.

REFERENCES

[1] PSEP-4 – Draft document by the Instrument Evaluation Subcommittee, Evaluation Protocols Area, National Committee for Clinical Laboratory Standards, 771 E.Lancaster Avenue, Villanova, PA 19085, 1978.

[2] Barbour, H.M., Virapen, K., Woods, T.F. and Burnett, D. An Evaluation of the Kodak Glucose/BUN Analyzer including experience with the NCCLS Protocol, PSEP-2, 3 and 4, *Journal of Automatic Chemistry,* this issue, 173.

[3] Davis, R.B., Thompson, J.E. and Pardue, H.L. (1978), *Clinical Chemistry,* **24**, 611.

[4] Westgard, J.D. and Hunt, M.R. (1973), *Clinical Chemistry,* **19**, 49.

[5] Westgard, J.D., de Vos, D.J., Hunt, M.R., Quam, E.F., Garber, C.C. and Carey, R.N. (1978), *American Journal of Medical Technology,* **44**, 552.

ACKNOWLEDGEMENTS

The authors would like to acknowledge the help of Kodak in supplying instruments and materials used in this investigation. We have valued comment from Drs R.B. Coolen and S. Reynolds and extensive secretarial help from Mrs Rosemary Jones during the preparation of this paper.

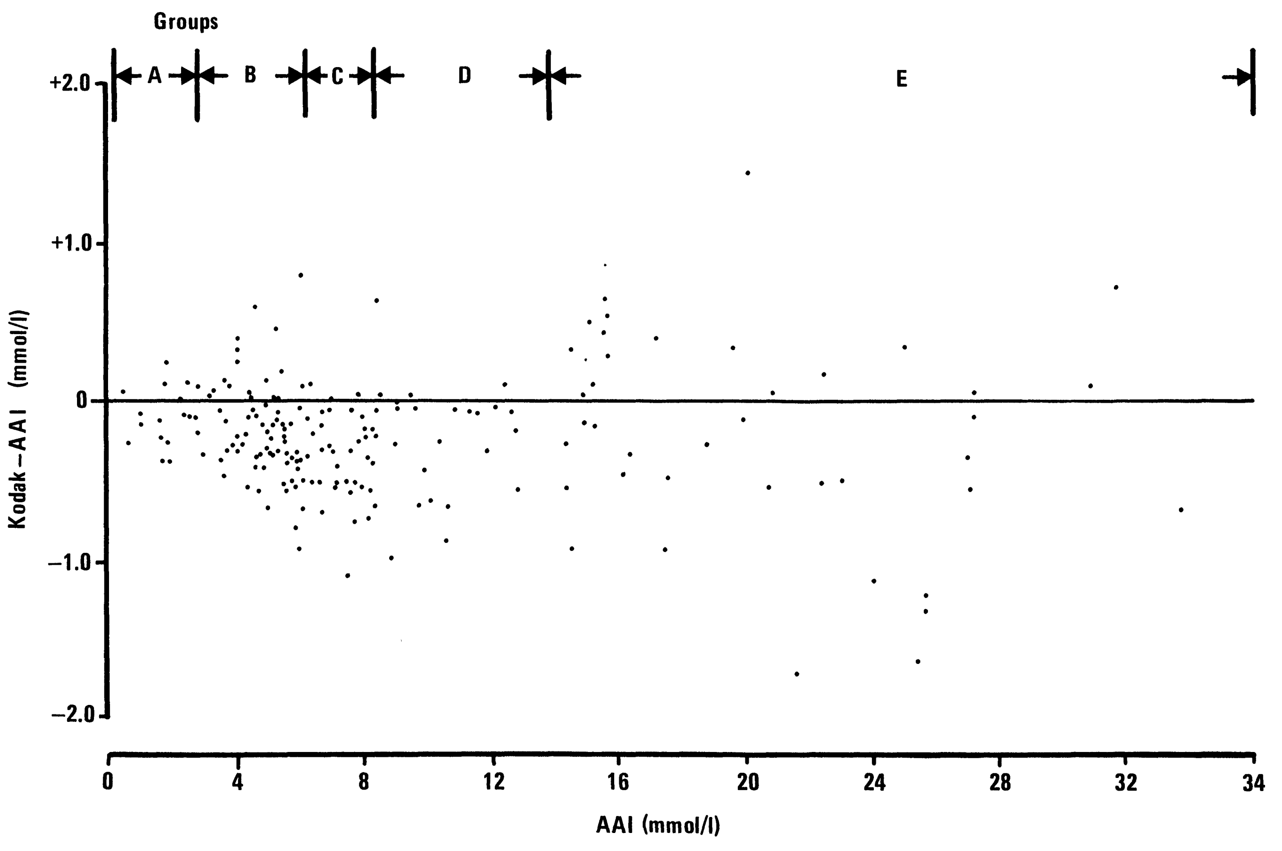

*Figure 1. Glucose comparative method (X) and Kodak Ektachem-Comparative method (Y) for patient samples Groups 'A' to 'E' as specified in text.*

